# General Public Knowledge, Attitude, and Practice Regarding the Impact of Air Pollution and Cardiopulmonary Diseases in Jeddah, Saudi Arabia

**DOI:** 10.7759/cureus.48976

**Published:** 2023-11-17

**Authors:** Nawaf A Alahmadi, Rakan Alzahrani, Abdullatif G Bshnaq, Mohammed A Alkhathlan, Abdulrahman A Alyasi, Abeer M Alahmadi, Muhammad A Khan, Syed Faisal Zaidi

**Affiliations:** 1 Medicine, King Saud Bin Abdulaziz University for Health Sciences, Jeddah, SAU; 2 Research and Development, King Abdullah International Medical Research Center, Jeddah, SAU; 3 Family Medicine, Program of Postgraduate Studies in Family Medicine, Public Health Administration, Ministery of Health, Jeddah, SAU; 4 Medical Education, King Saud Bin Abdulaziz University for Health Sciences, Jeddah, SAU; 5 Faculty of Eastern Medicine, Hamdard University, Islamabad, PAK

**Keywords:** covid-19, saudi arabia, jeddah, perception, knowledge, awareness, pulmonary diseases, cardiovascular diseases, outdoor air pollution, indoor air pollution

## Abstract

Introduction

Air pollution is a critical public health issue associated with various respiratory and cardiovascular diseases. The lungs and heart are the organs most affected by air pollution, and damage to these organs is strongly associated with inhaled particulate matter produced by burning fossil fuels. Household and ambient air pollution have been closely linked to lower respiratory infections, with ambient air pollution alone estimated to be responsible for millions of deaths globally each year. Therefore, this study aimed to assess the general public knowledge attitude and practice regarding air pollution and cardiopulmonary morbidity in Jeddah, Saudi Arabia.

Methods

The study was conducted in Saudi Arabia using a self-administered questionnaire distributed through popular social media apps. A snowball sampling technique was used, including only Saudi citizens aged 18 or older. The questionnaire consisted of 30 questions derived from a comprehensive literature review on the subject matter. Questions were validated through face validity, pilot testing, and Cronbach's alpha reliability measurement. The questionnaire included questions on demographic data, knowledge of air pollution, the relationship between air pollution and cardiopulmonary diseases, and attitudes and practices toward lowering exposure to air pollution.

Results

The study included 649 participants, with a mean age of 32.11 ± 13.47 years, and over half were females (54.7%). Most participants were aware of outdoor and indoor air pollution, but only a tiny percentage recognized cooking as a primary indoor source of pollution. However, the majority believed that indoor pollution could contribute to outdoor pollution. Participants associated air pollution with cardiopulmonary diseases, mainly secondhand tobacco smoke and outdoor air pollution caused by factories and industrial facilities. Knowledge and practice levels varied, with older individuals, females, and those in non-health-related occupations having higher levels of knowledge. Positive attitudes, particularly believing that moving to a less polluted area improves health, were associated with better knowledge. Females exhibited better air pollution-related practices, and there was a positive correlation between knowledge and practice scores.

Conclusion

The study highlighted the need for targeted public health campaigns to improve awareness and promote healthier practices, particularly among young adults, to mitigate the potential health impacts of air pollution, especially cardiopulmonary health.

## Introduction

Air pollution is a mixture of gasses, liquids, and solids in the air, the sum of which is called particulate matter (PM) [1]. PM are small hazardous particles of differing origins and properties, such as smoke, dust, pollen, and soot, and they are commonly associated with health risks [2]. A more substantial exposure to PM caused by either an increased dose of PM or increased exposure time directly increases the amount of PM that reaches relevant organs where dangerous effects can occur [3]. For example, the lungs and the heart are the organs most affected by this, and the damage to them is strongly associated with the inhaled PM produced by burning fossil fuels, a common source of pollution in metropolitan cities [4]. Fossil fuels are also a significant source of PM 2.5 (PM with a size of 2.5 micrometers and lower). PMs of this size can penetrate the lungs' alveoli deeply and irritate its walls, damaging the lungs and disrupting their function [2].

Both household and ambient air pollution have been closely associated with lower respiratory infections. Ambient air pollution alone is estimated to be responsible for 8.8 million deaths per year globally, with a loss of life expectancy of 2.9 (2.3-3.5) years [5]. A study conducted in China showed that, during the 2003 severe acute respiratory syndrome (SARS) epidemic, regions with moderate air pollution index (APIs) that derived from the concentrations of PM had an 84% increased risk of mortality rate to SARS than regions with lower APIs from SARS compared to those from regions with low API [6].

Furthermore, emerging evidence on COVID-19 suggests a positive between exposure to air pollution and the spread of the virus and the mortality rate [7,8]. A study of the global disease burden in 2016 suggests that interventions to lowering both household air pollution or indoor air pollution (IAP) and ambient air pollution could be vital in lowering the mortality rate of lower respiratory infection [9]. In the current COVID-19 pandemic, masses were advised to stay isolated in their residential spaces, increasing the chances of IAP's impact on their health. Moreover, inadequate ventilation inside residential areas can further augment the impact of IAP, particularly on patients suffering from cardiopulmonary disorders [10]. Assessing and increasing awareness regarding air pollution and PM size could be an effective intervention. Not only could these interventions be of possible help in dealing with the spread of COVID-19, but they could also help lower the baseline demand on hospitals for other respiratory infections and diseases associated with air pollution [11].

Ambient air pollution is a serious environmental issue in the Gulf Cooperation Council (GCC) countries, including Saudi Arabia [12]. The global rankings of all the GCC countries in relation to their PM2.5 concentrations are 140 or higher on the environmental performance index. Saudi Arabia is considered a country that suffers from outdoor air pollution [13].

To date, no study has assessed general public awareness of the impact of air pollution (both indoor and outdoor) on the Saudi population. Therefore, this study is directed toward the general public of Jeddah, Saudi Arabia, to examine their level of perception and awareness towards air pollution.

## Materials and methods

This study was conducted in Saudi Arabia by survey through popular social media apps (Instagram, Twitter, and WhatsApp). Saudi Arabia is considered the largest country in the Middle East. The Kingdom's population was estimated in 2020 to be approximately 35 million, according to the Unified National Platform of Saudi Arabia. The number of internet users in Saudi Arabia was estimated to be 19.6 million in 2018, constituting 58% of the population.

The study utilized a self-administered questionnaire published through various media outlets from the 31st of July to the 10th of August 2022. The platform used to construct the questionnaire was Google Forms. A snowball sampling technique was used, in which the participants were encouraged to spread the questionnaire to their friends and family. The participants included only Saudi citizens who were 18 or older. Informed consent was obtained from all participants. Participants who did not meet our inclusion criteria or did not complete the questionnaire were excluded from the study. Additionally, the participants were limited to one submission to eliminate response bias. An online survey was chosen to collect responses due to the limitations of the ongoing COVID-19 pandemic.

The questionnaire consisted of questions derived from an extensive literature review on the subject. The questionnaire was validated by face validity from an expert in medical education. The clarity of the questions was checked through a pilot study, and the reliability was measured by calculating Cronbach's alpha, which was 0.81. Language experts checked the Arabic translation. The questions were then edited based on the feedback from pilot testing. Each question had a particular score assigned to it. The cumulative points were then put into a scoring system to obtain the total knowledge and practice score. The questionnaire given to the subjects consists of seven questions on demographic data. Then, the second section included 31 questions assessing knowledge of air pollution, nine questions about practices related to lowering exposure to air pollution, and one question to assess participants toward the attitudes regarding air pollution. The total knowledge score was 41.

Based on the total score, participants were then divided into three categories: adequate (good), average (fair), and inadequate (poor) for both knowledge and practice. A total score of 75% correct answers and above were categorized as adequate, a score between 65 and 75 was considered average, and a score below 65% was considered inadequate knowledge or practice [14].

The institutional review board (IRB) at King Saud bin Abdelaziz University for Health Sciences approved this study ethically with an ethical approval number: SP21J/103/03. The participants signed an informed consent before the questionnaire. No identifying data, such as names or IDs, were taken from the participants. Participants' responses were identified with assigned numbers only. All participants' information was kept secure and password-protected, and only the researchers had access to the information.

Data analysis

Data were analyzed using the Statistical Product and Service Solutions (SPSS, version 26) (IBM SPSS Statistics for Windows, Armonk, NY) program. The chi-squared test (χ2) was used for categorical data that were expressed as numbers and percentages to assess the association between the variables. Quantitative data were presented as mean and standard deviation (mean ± SD), where the Kruskal-Wallis test was applied for non-parametric variables. Correlation analysis using Spearman's test was done, and a p-value of <0.05 was considered statistically significant.

## Results

The mean age of the studied 649 participants was 32.11 ± 13.47 years (Table 1). Specifically, 54.7% were females, 56.9% were single, and 64.7% had a bachelor's degree in education. About 11 (11.9%) were working in a health-related occupation. Of them, 16.8% had chronic diseases, and 8.6% had cardiopulmonary diseases, with asthma being the most common (84.3%).

**Table 1:** Distribution of studied participants according to their demographic characters (N: 649)

Variable	N (%)
Age (years) (mean ± SD)	32.11 ± 13.47
Gender	
Male	355 (54.7)
Female	294 (45.3)
Marital status	
Married	248 (38.2)
Divorced	20 (3.1)
Widow	12 (1.8)
Single	369 (56.9)
Educational level	
Less than high school	9 (1.4)
High school	171 (26.3)
Bachelor degree	420 (64.7)
Postgraduate education	49 (7.6)
Occupation	
Heath related	77 (11.9)
Non-health related	244 (37.6)
Unemployed	55 (8.5)
Housewife	56 (8.6)
Student	217 (33.4)
Having a chronic disease	
Yes	109 (16.8)
No	540 (83.2)
Do you have any cardiopulmonary diseases?	
Yes	56 (8.6)
No	593 (91.4)
If having a cardiopulmonary disease, what is it: (N: 56)	
Heart attack	2 (3.5)
Atherosclerosis	4 (7.1)
Asthma	46 (84.3)
Mitral valve regurgitation and prolapse	1 (1.7)
atrial fibrillation	1 (1.7)
Lung infection	1 (1.7)

Most participants (84.9% and 62.7%) knew the meaning of outdoor and indoor air pollution, respectively. Of them, only 8.3% considered cooking the primary cause of indoor air pollution. However, 70.3% thought indoor air pollution could cause outdoor air pollution. The majority (81.5%) thought pollutants contaminated the air around them, and 57.2% scaled the pollution around them as moderate. Most of them (88%) knew the causes of outdoor air pollution, and the most common pollutants that they thought contributed to outdoor air pollution were factories (87.4%) and industrial facilities. About 7% (67.2%) knew the causes of indoor air pollution, and the most common pollutants that they thought to contribute to outside air pollution were smoking (94.1%), pesticides (78%), and starting car in closed garage (61.9%) (Table 2).

**Table 2:** Distribution of the studied participants according to their knowledge about air pollution (N: 649)

Variable	Yes N (%)	No N (%)
Do you understand the meaning of outdoor air pollution?	551 (84.9)	98 (15.1)
Do you understand the meaning of indoor air pollution?	407 (62.7)	242 (37.3)
Would you consider cooking to be the major cause of indoor air pollution?	54 (8.3)	595 (91.7)
Do you think indoor Air Pollution could cause outdoor air pollution?	456 (70.3)	193 (29.7)
Do you think the air around you is contaminated by pollutants?	529 (81.5)	120 (18.5)
If so, how much would you scale the pollution around you? (N: 529)		
Low	159 (30)	
Moderate	203 (57.2)	
Sever	68 (12.8)	
Know the causes of outdoor air pollution	571 (88)	
Which of these do you think contributes to outside air pollution? (more than one option can be selected)		
Factories	567 (87.4)	
Fossil fuels	316 (48.7)	
Industrial facilities	466 (71.8)	
Dust particles	406 (62.6)	
Power plants	306 (47.4)	
Oil refinery	395 (60.9)	
Know the causes of indoor air pollution	436 (67.2)	
Which of these do you think contributes to Indoor air pollution? (more than one option can be selected)		
Smoking	611 (94.1)	
burning incense *Bakhor*	256 (39.4)	
Mold	374 (57.6)	
Cleaning products	328 (50.5)	
Pesticides	506 (78)	
Pets	238 (36.7)	
Heating devices	131 (20.2)	
Candle use	179 (27.6)	
Uncleaned furniture	324 (50)	
Starting the car in a closed garage	401 (61.9)	

Most participants (94.8%) believed that air pollution could be a causality of cardiopulmonary disease, and 83.2% thought that air pollution contributes to asthma or other diseases such as COVID-19. The main sources of the participants' belief that air pollution could cause adverse effects on their health were social media (55.6%) and articles (52.7%) (Table 3).

**Table 3:** Participants' knowledge about the association of air pollution with cardiopulmonary diseases and sources of information on the effect of air pollution on their health (N: 649)

Variable	N (%)
Do you believe that air pollution could be a causality of cardiopulmonary disease?	
Yes	615 (94.8)
No	34 (5.2)
In your opinion, does air pollution contribute to asthma or other diseases like COVID-19	
Yes	450 (83.2)
No	109 (16.8)
How did you find out that air pollution could cause an adverse effect on your health?	
Social media	361 (55.6)
Friends	181 (27.9)
Scientific published articles	342 (52.7)
Self-experience	295 (45.5)

The most common factors that were highly agreed (proven) to be related to cardiovascular or pulmonary diseases were secondhand tobacco smoke (68.3%) and outdoor air pollution (e.g., factories and automobiles) (51.6%) (Table 4).

**Table 4:** Participants' knowledge about factors related to cardiovascular or pulmonary diseases (N: 649)

Variable	High (proven, agree) N (%)	Probably (likely) N (%)	Medium (somewhat likely) N (%)	Not likely (Doubt a relationship) N (%)	
					Do you think the following is related to cardiovascular or pulmonary diseases?					
Secondhand tobacco smoke	443 (68.3)	157 (24.2)	43 (6.6)	6 (0.9)	
					Global climate change	163 (25.1)	291 (44.8)	133 (20.5)	62 (9.6)	
Outdoor air pollution (e.g., factories, automobiles)	335 (51.6)	242 (37.3)	64 (9.9)	8 (1.2)						
					Extreme temperatures	126 (19.4)	235 (36.2)	182 (28)	106 (16.3)	
Indoor air pollution	169 (26)	246 (37.9)	183 (28.2)	51 (7.9)						

**Figure 1:**
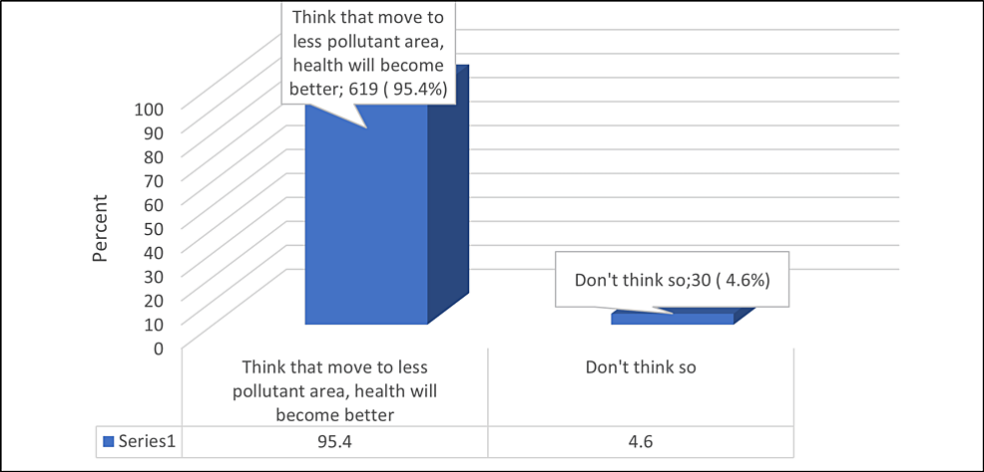
Percentage distribution of the participants' attitude toward lowering exposure to air pollution

As for the participants' attitude toward lowering exposure to air pollution, the majority (95.4%) thought that moving to a less pollutant area would improve their health (Figure 1).

Regarding the participants' practice, 84.4% were cooking daily, and 95.1% had good health before they started cooking. Most of them (93.1%) used measures to ventilate the air while cooking with an exhaust fan (92.3%), the most commonly used measure. About 40% (40.8%) tried any ways to lower their exposure to outdoor air pollution, while 50.8% tried ways to indoor air pollution (Table 5).

**Table 5:** Participants' practices regarding lowering exposure to air pollution (N: 649)

Variable	N (%)
Cooking experience	
Do you cook on a daily basis?	
Yes	548 (84.4)
No	101 (15.6)
If yes, how was your health before you start cooking (N.: 548)	
Well	521 (95.1)
Not well	27 (4.9)
Practices toward lowering exposure to air pollution	
Do you use measures to ventilate the air while cooking?	
Yes	604 (93.1)
No	45 (6.9)
If yes which of these methods, do you use? (N.: 604)	
Open kitchen window	386 (63.9)
Exhaust fan	558 (92.3)
Exhaust hood	248 (41)
Open kitchen doors	309 (51.1)
Have you ever tried any ways to lower your exposure to outdoor air pollution?	
Yes	256 (40.8)
No	384 (59.2)
Have you ever tried any ways to lower your exposure to indoor air pollution?	
Yes	330 (50.8)
No	319 (49.2)

**Figure 2:**
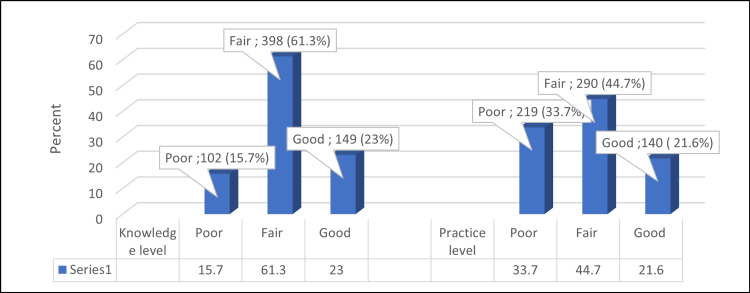
Percentage distribution of the levels of knowledge and practice of the participants' regarding air pollution

The mean knowledge score was 26.27 ± 5.73, and the mean practice score was 4.16 ± 1.59. As for the participants' knowledge about air pollution, 23% had good knowledge, and 61.3% and 15.7% had fair and poor levels, respectively. Regarding their practice, 21.6% had a good practice level, 44.7% had a fair practice, and 33.7% had a poor practice (Figure 2).

Table 6 shows that good knowledge about air pollution was significantly higher among participants with older mean age, females, married, and those with a non-health related occupation (p = <0.05).

**Table 6:** Relationship between participants' knowledge about air pollution and their demographic characters (N: 649) N.B.: * = Kruskal Wallis test

Variable	Knowledge level			χ2	p-value
	Poor N. (%)	Fair N. (%)	Good N. (%)		
Age (mean ± SD)	26.94 ± 11.02	31.63 ± 13.42	36.91 ± 13.56	2*	<0.001
Gender					
Male	80 (78.4)	212 (53.3)	63 (42.3)	32.78	<0.001
Female	22 (21.6)	186 (46.7)	86 (57.7)		
Marital status					
Married	24 (23.5)	144 (36.2)	80 (53.7)	29.19	<0.001
Divorced	2 (2)	12 (3)	6 (4)		
Widow	1 (1)	9 (2.3)	2 (1.3)		
Single	75 (73.5)	233 (58.5)	61 (40.9)		
Educational level					
Less than high school	1 (1)	4 (1)	4 (2.7)	5.23	0.514
High school	25 (24.5)	113 (28.4)	33 (22.1)		
Bachelor degree	70 (68.6)	251 (63.1)	99 (66.4)		
Postgraduate education	6 (5.9)	30 (7.5)	13 (8.7)		
Occupation					
Heath related	12 (11.8)	45 (11.3)	20 (13.4)	24.56	0.002
Non-health related	29 (28.4)	149 (37.4)	66 (44.3)		
Unemployed	13 (12.7)	32 (8)	10 (6.7)		
Housewife	3 (2.9)	33 (8.3)	20 (13.4)		
Student	45 (44.1)	139 (34.9)	33 (22.1)		
Having a chronic disease					
Yes	13 (12.7)	69 (17.3)	27 (18.1)	1.46	0.48
No	89 (87.3)	329 (82.7)	122 (81.9)		
Do you have any cardiopulmonary disease diseases?					
Yes	4 (3.9)	34 (8.5)	17 (11.4)	4.38	0.112
No	98 (96.1)	364 (91.5)	132 (88.6)		

**Figure 3:**
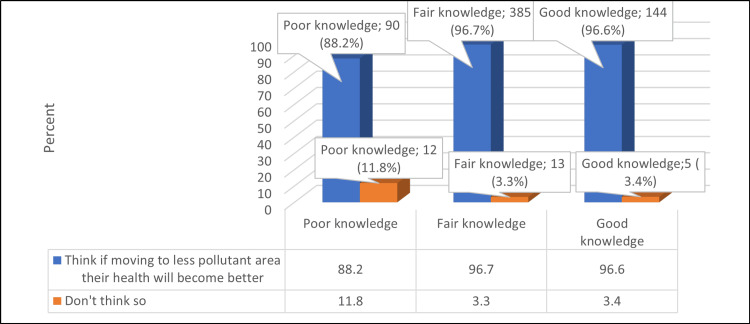
Relationship between participants' knowledge about air pollution and their attitude N.B.: (χ2 = 14, p-value = 0.001)

Figure 3 illustrates that good knowledge about air pollution was significantly higher among participants with positive attitudes who thought that if they moved to less pollutant areas, their health would improve (p = <0.05).

**Figure 4:**
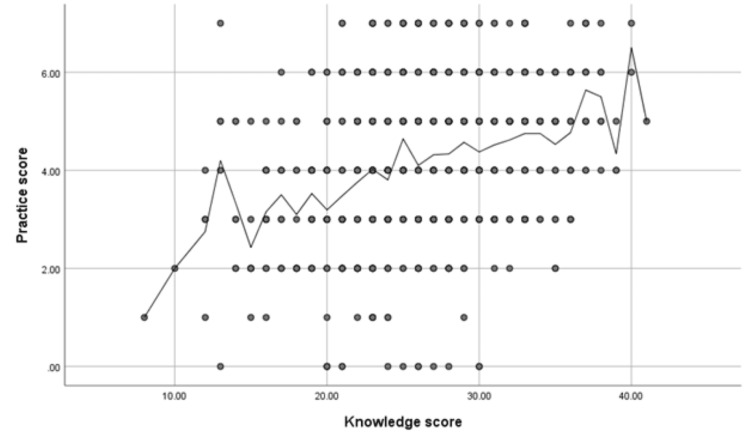
Spearman's correlation analysis between knowledge scores and practice scores N.B.: (r = 0.32, p-value = <0.001)

Table 7 demonstrates that good practice related to air pollution was significantly higher among females (p = <0.05). On the other hand, a non-significant relationship was found between participants' practice and all other demographics (p = >0.05). A significant positive correlation was found between knowledge scores about air pollution and practice scores (r = 0.32, p-value = <0.001) (Figure 4).

**Table 7:** Relationship between participants' practice related to air pollution and their demographic characters (N: 649)

Variable	Practice level			χ2	p-value
	Poor N (%)	Fair N (%)	Good N (%)		
Age (Mean ± SD)	30.11 ± 12.81	32.81 ± 13.47	33.78 ± 14.21	2.14*	0.128
Gender					
Male	146 (66.7)	146 (50.3)	63 (45)	20.19	<0.001
Female	73 (33.3)	144 (49.7)	77 (55)		
Marital status					
Married	76 (34.7)	112 (38.6)	60 (42.9)	9.01	0.173
Divorced	2 (0.9)	13 (4.5)	5 (3.6)		
Widow	4 (1.8)	5 (1.7)	3 (2.1)		
Single	137 (62.6)	160 (55.2)	72 (51.4)		
Educational lev					
Less than high school	3 (1.4)	3 (1)	3 (2.1)	2.66	0.849
High school	63 (28.8)	74 (25.5)	34 (24.3)		
Bachelor degree	138 (63)	188 (64.8)	94 (67.1)		
Postgraduate education	15 (6.8)	25 (8.6)	9 (6.4)		
Occupation					
Heath related	29 (13.2)	34 (11.7)	14 (10)	8.71	0.367
Non-health related	72 (32.9)	112 (38.6)	60 (42.9)		
Unemployed	19 (8.7)	26 (9)	10 (7.1)		
Housewife	14 (6.4)	30 (10.3)	12 (8.6)		
Student	85 (38.8)	88 (30.3)	44 (31.4)		
Having a chronic disease					
Yes	34 (15.5)	50 (17.2)	25 (17.9)	0.4	0.816
No	185 (84.5)	240 (82.8)	115 (82.1)		
Do you have any cardiopulmonary disease diseases?					
Yes	17 (7.8)	26 (9)	12 (8.6)	0.23	0.889
No	202 (92.2)	264 (91)	128 (91.4)		
Do you think if you move to less pollutant area your health will become better?					
Yes	204 (93.2)	278 (95.9)	137 (97.9)	4.57	0.102
No	15 (6.8)	12 (4.1)	3 (2.1)		

## Discussion

The main finding of this study was that the majority of participants understood the concept of outdoor and indoor air pollution, with cooking being the most common cause. Most of them (81.5%) believed the air was polluted, with factories and industrial facilities being the main contributors. Indoor air pollution was mainly caused by smoking, pesticides, and starting cars in closed garages. Most participants (94.8%) believed that air pollution could be a causality of cardiopulmonary disease, and 83.2% thought that air pollution contributes to asthma or other diseases like COVID-19. The primary sources of the participants' belief that air pollution could cause adverse effects on their health were social media (55.6%) and articles (52.7%). The most common factors that were high (proven) to be related to cardiovascular or pulmonary diseases were secondhand tobacco smoke (68.3%) and outdoor air pollution (e.g., factories and automobiles) (51.6%). As for the participants' attitude toward lowering exposure to air pollution, the majority (95.4%) thought that moving to less pollutant areas would improve your health.

Regarding the participants' practice, 84.4% were cooking daily, and 95.1% had good health before they started cooking. Most of them (93.1%) used measures to ventilate the air while cooking with an exhaust fan (92.3%), the most commonly used measure. About 40%-40.8% tried any ways to lower their exposure to outdoor air pollution, while 50.8% tried ways for indoor air pollution.

Participants' source of information

In this study, social media (55.6%) and articles (52.7%) were the primary sources of participants' belief that air pollution could harm their health. In the same vein, a study conducted in Oman discovered that most participants preferred social media to other sources of information about air pollution, such as newspapers, TV, and radio. Furthermore, authors discovered that specific factors, such as female sex, middle age groups, and bachelor's degree holders use social media significantly more than their counterparts [15]. Furthermore, in a study conducted in Wuhan, China, 57% of participants used mobile phone applications to access air quality information [16]. These findings indicate that social media is the primary source of information about air pollution in Saudi Arabia for the general public. This channel is critical for disseminating air pollution information and may be important in informing the public about environmental pollution in real time. As a result, authorities can use social media platforms to inform the public about outdoor air quality, particularly in places with more vulnerable people, such as schools, children's play yards, and elderly care centers.

Participants’ knowledge

The mean knowledge score was 26.27 ± 5.73, 23% had a good level of knowledge, and 61.3% and 15.7% had a fair and a poor level. Good knowledge about air pollution was significantly higher among participants with older mean age, females, married, and those with a non-health related occupation. Our results are consistent with the body of literature [16,17]. Larijani reported that, among 300 teachers (136 male and 164 female), females had significantly higher levels of environmental awareness than males [17]. AlShidi et al. found that, among 1,289 participants (34% were females), the level of knowledge was significantly higher among females compared to males [15]. Regarding age, older age participants were found to have a higher level of knowledge. This finding was consistent with Fischer et al. [18]. On the other hand, Unni et al. found that those aged 20-29 and 30-39 had the highest levels of knowledge. They reported a declining trend in knowledge with age [19]. The inconsistent direction of age's effect on knowledge levels could be attributed to cultural and environment-specific differences in knowledge acquisition across settings. For example, younger segments of some populations may have greater access to information and news through increased internet usage. In contrast, older people with higher potential pollutant exposure may be more proactive in understanding possible adverse health outcomes caused by such exposure. In this study, we did not find a significant association between the education level and knowledge level. A similar finding was reported by Unni et al. [19]. On the other hand, many studies reported a significant association between the level of education and knowledge about environmental pollution [17,19,20]. According to one international study, educational level is one of the top three predictors of climate change awareness [21]. More research is needed to understand the unpredictable effects of education qualification levels on knowledge, including the role of health literacy.

We found a significant association between occupation and level of knowledge. A cross-sectional study in Nanjing, China, reported that white-collared occupations had higher knowledge scores compared to those in blue-collared ones, with the study investigators hypothesizing those in higher-skilled roles might have had access to more resources and health-related information, possibly resulting in higher knowledge scores compared to their lower skilled counterparts [22].

Participants' practice

Regarding their practice, the mean practice score was 4.16 ± 1.59. Moreover, 21.6% had a good practice level, 44.7% had a fair practice, and 33.7% had a poor one. Good practice related to air pollution was significantly higher among females. On the other hand, a non-significant relationship was found between participants' practice and all other demographics. Different sociodemographic factors associated with good practice were addressed in the literature, including willingness to pay to save the environment [23], country income level [24], and occupation [25]. We found a significant positive correlation was found between knowledge scores about air pollution and practice scores. The association between knowledge and behavior was broadly consistent with findings from other studies that assessed attitude and behavior (KAB) toward environmental issues in a global population and studies that assessed KAB toward various health issues in the Singapore population [19,26,27].

Strengths and limitations

This study's originality and contribution to the existing knowledge base are significant strengths. No research has been conducted to assess the general public's awareness of the impact of air pollution on the Saudi population. By filling this gap, the study offers invaluable insights that can be used to inform public health interventions and policies. Furthermore, the questionnaire's extensive validation process, including face validity, content validity, and pilot testing, ensures that the data collected are reliable and valid. One notable limitation of this study is the use of a snowball sampling technique. While this method is practical, it may introduce selection bias because participants may refer individuals with similar views or backgrounds, potentially resulting in a non-representative sample. Additionally, the exclusion of non-Saudi citizens may limit the sample's diversity and the findings' generalizability.

## Conclusions

Most participants were aware of outdoor and indoor air pollution but did not recognize cooking as a primary indoor pollution source. Interestingly, many believed indoor pollution could contribute to outdoor pollution. Participants believed the air around them was contaminated, mainly attributed to factories and industrial facilities. They associated air pollution with cardiopulmonary diseases, influenced by information from social media and articles. Secondhand tobacco smoke and outdoor air pollution were perceived as major factors causing cardiovascular and pulmonary diseases. In terms of practices, participants frequently engaged in cooking activities and used ventilation measures such as exhaust fans. A substantial portion attempted to mitigate outdoor and indoor air pollution exposure. Knowledge and practice levels varied, with older individuals and those in non-health-related occupations having higher knowledge levels. Positive attitudes, particularly believing that moving to a less polluted area improves health, were associated with better knowledge. These findings emphasize the need for targeted public health campaigns to improve awareness and promote healthier practices, particularly among young adults, to mitigate the potential health impacts of air pollution, especially cardiopulmonary health.

## Appendices

Questionnaire used in this study

**Table 8:** Demographical data

Demographical data
1. Gender
□ Male □ Female
2. Age
…………. years
3. Marital status:
□ Married □ Divorced □ Widow □ Single
4. Educational level:
□ Illiterate □ Less than high school □ High school □ Bachelor degree □ Post-graduate education
5. Occupational status:
□ Health related □ Non-health-related □ Unemployed □ Housewife
6. Do you suffer from any chronic disease?
□ Yes □ No
If yes please mention it: ……………………………

**Table 9:** Knowledge on air pollution

Knowledge on air pollution
1. Do you know the meaning of outdoor air pollution?
□ Yes □ No
2. Do you know the meaning of indoor air pollution?
□ Yes □ No
3. Do you think the air around you is contaminated by pollutants?
□ Yes □ No
4. If so, how much would you scale the pollution around you?
□ Low □ Moderate □ Severe
5. Do you know the causes of outdoor air pollution?
□ Yes □ No
6. Which of these do you think contributes to outside air pollution? (more than one option can be selected)
□ Factories □ Fossile fuels □ Industrial facilities □ Dust particles □ Power plants □ Oil refinery
7. Do you know the causes of indoor air pollution?
□ Yes □ No
8. Which of these do you think contributes to indoor air pollution? (more than one option can be selected)
□ Smoking □ Burning incense (Bakhor) □ Mold □ Cleaning products □ Pesticides □ Pets □ Heating devices □ Candle use □ Uncleaned furniture □ Starting car in closed garage
9. Would you consider cooking to be the major cause of indoor air pollution?
□ Yes □ No
10. Do you think indoor air pollution could cause outdoor air pollution?
□ Yes □ No

**Table 10:** Knowledge about the association of air pollution with cardiopulmonary diseases

Knowledge about the association of air pollution with cardiopulmonary diseases
1. Do you have any cardiopulmonary disease disease?
□ Yes □ No
2. If yes what is it:
□ Heart failure □ Heart attack □ Angioplasty □ Asthma □ COPD □ Other …………………………………
3. Do you believe that air pollution could be a cause of cardiopulmonary disease?
□ Yes □ No
4. Which of these factors do you think are related to cardiovascular or pulmonary diseases?
High (proven) Probably (likely) Medium (somewhat likely, but not proven) Not likely (doubt a relationship) Secondhand tobacco smoke Global climate change Outdoor air pollution (eg, factories automobiles) Extreme temperatures Indoor Air Pollution
5. In your opinion, does air pollution contribute to asthma or other diseases like COVID-19?
□ Yes □ No
6. How did you find out that air pollution could cause adverse effect on your health?
□ Social media □ Friends □ scientific Articles □ Self experience □ other …………………………
7. Do you think if you move to a less pollutant area your health will become better?
□ Yes □ No

**Table 11:** Attitude and practices toward lowering exposure to air pollution

Attitude and practices toward lowering exposure to air pollution
1. Do you cook on a daily or weekly basis?
□ Yes □ No
2. How was your health before you start cooking
□ Well □ Not well
3. Do you use measures to ventilate the air while cooking?
□ Yes □ No
4. If yes which of these methods do you use?
□Open kitchen’s window □Exhaust fan □Exhaust hood □Open kitchen’s doors □ Other …………..
5. Have you ever tried any ways to lower your exposure to outdoor air pollution?
□ Yes □ No
6. Have you ever tried any ways to lower your exposure to indoor air pollution?
□ Yes □ No
